# Novel Retro-Inverso Peptide Antibiotic Efficiently Released by a Responsive Hydrogel-Based System

**DOI:** 10.3390/biomedicines10061301

**Published:** 2022-06-02

**Authors:** Angela Cesaro, Rosa Gaglione, Marco Chino, Maria De Luca, Rocco Di Girolamo, Angelina Lombardi, Rosanna Filosa, Angela Arciello

**Affiliations:** 1 Machine Biology Group, Departments of Psychiatry and Microbiology, Institute for Biomedical Informatics, Institute for Translational Medicine and Therapeutics, Perelman School of Medicine, University of Pennsylvania, Philadelphia, PA 19104, USA; angela.cesaro@pennmedicine.upenn.edu; 2 Departments of Bioengineering and Chemical and Biomolecular Engineering, School of Engineering and Applied Science, University of Pennsylvania, Philadelphia, PA 19104, USA; 3 Penn Institute for Computational Science, University of Pennsylvania, Philadelphia, PA 19104, USA; 4 Department of Chemical Sciences, University of Naples Federico II, Via Cintia 21, I-80126 Naples, Italy; rosa.gaglione@unina.it (R.G.); marco.chino@unina.it (M.C.); maria.deluca2@unina.it (M.D.L.); rocco.digirolamo@unina.it (R.D.G.); alombard@unina.it (A.L.); 5 Istituto Nazionale di Biostrutture e Biosistemi (INBB), I-00136 Rome, Italy; 6 AMP Biotec, Research Start-Up, s.s. 7, km 256, I-82030 Apollosa, Italy; rfilosa@unisannio.it; 7 Department of Sciences and Technology, University of Sannio, Via De Sanctis, I-82100 Benevento, Italy

**Keywords:** drug design, antimicrobial peptidomimetic, hydrogel-based system, hyaluronic acid, anti-infective activity, skin infections

## Abstract

Topical antimicrobial treatments are often ineffective on recalcitrant and resistant skin infections. This necessitates the design of antimicrobials that are less susceptible to resistance mechanisms, as well as the development of appropriate delivery systems. These two issues represent a great challenge for researchers in pharmaceutical and drug discovery fields. Here, we defined the therapeutic properties of a novel peptidomimetic inspired by an antimicrobial sequence encrypted in human apolipoprotein B. The peptidomimetic was found to exhibit antimicrobial and anti-biofilm properties at concentration values ranging from 2.5 to 20 µmol L^−1^, to be biocompatible toward human skin cell lines, and to protect human keratinocytes from bacterial infections being able to induce a reduction of bacterial units by two or even four orders of magnitude with respect to untreated samples. Based on these promising results, a hyaluronic-acid-based hydrogel was devised to encapsulate and to specifically deliver the selected antimicrobial agent to the site of infection. The developed hydrogel-based system represents a promising, effective therapeutic option by combining the mechanical properties of the hyaluronic acid polymer with the anti-infective activity of the antimicrobial peptidomimetic, thus opening novel perspectives in the treatment of skin infections.

## 1. Introduction

The dramatic emergence of antibiotic resistance is leading to the design of novel antimicrobials that are less susceptible to resistance mechanisms than conventional antibiotics. In this context, innovative molecules such as antimicrobial peptides (AMPs) represent a promising alternative to the old generations of antibiotics since the evolution of resistance against these compounds is demonstrated to be less probable with respect to conventional antimicrobials [1,2,3,4]. Despite their numerous advantages, antimicrobial peptides’ conformation often makes them extremely sensitive to endo- and exopeptidases present in biological systems [5]. A powerful tool to transform proteolytically labile peptides into compounds with improved bioactivities and pharmacokinetic profiles is represented by the development of peptidomimetics. Peptidomimetics are defined as “*compounds whose essential elements mimic a natural peptide and that retain the ability to interact with the biological target by producing the same biological effect*” [6]. Many different approaches are used to generate peptidomimetics, and the selection of the most suitable design strategy depends on the available information about the parental peptide in terms of structure, sequence, and function [6,7]. To ensure peptide proteolytic stability, we modified the primary structure of a naturally occurring antimicrobial peptide encrypted in human plasma apolipoprotein B [8,9,10,11,12,13,14,15,16,17] by designing a retro-inverso sequence composed entirely of D-enantiomeric amino acids, here named (ri)-r(P)ApoB_S_^Pro^ [17]. In the present work, we investigate and characterize the biological activities of the designed peptidomimetic and develop a suitable topical delivery system, allowing the specific release of the selected antimicrobial in therapeutic procedures.

Several formulations, including suspensions, micro/nanoparticles, patches, and hydrogels, have been proposed to control topical drug delivery. Among different alternatives, hydrogel-based systems have been found to offer many advantages in the treatment of skin wounds and infections [18,19]. According to the definition, hydrogel-based systems are networks of natural or synthetic cross-linked polymers with a three-dimensional configuration able to imbibe high amounts of water [20]. The hydrogel’s swelling ability is the result of the hydrophilic moieties in the structure, which are resistant to water dissolution thanks to the presence of cross-linkers between polymeric chains [21]. Several polymers have been chemically modified to develop synthetic hydrogels with improved properties [21]. These delivery systems are classified on the basis of their origin, physical properties, nature of swelling, method of preparation, ionic charges, rate of biodegradation, and kind of cross-linking [22]. Recently, hydrogels based on hyaluronic acid (HA) confirmed their excellent properties not only as scaffolds but also as suitable carriers of biologically active substances [20]. HA is an essential component of the natural extracellular matrix, where it plays an important role in many biological processes, including wound healing. HA is biocompatible, biodegradable, and non-immunogenic and has unique viscoelastic and rheological properties that make it an excellent polymer to build hydrogel systems with desired morphology and bioactivity [23]. Here, we developed and characterized an HA-based hydrogel system loaded with the peptidomimetic (ri)-r(P)ApoB_S_^Pro^ with the main aim to specifically deliver this antimicrobial agent to the site of infection. We show that the combination of HA biological properties, of hydrogel system swelling and hydrating capabilities, together with the efficacy of the selected antimicrobial peptidomimetic concurs to create a promising and effective system that is able to target skin disorders and infections, thus opening interesting perspectives to the future applicability of the retro-inverso peptide in the dermatological field.

## 2. Materials and Methods

### 2.1. Materials

All the reagents were purchased from Sigma-Merck (Milan, Italy), unless differently specified. HA cross-linked with 2.5% 1,4-butanediol diglycidyl ether (BDDE) was provided by Altergon Italia s.r.l. (Morra De Sanctis, AV, Italy).

### 2.2. Peptide Synthesis

(ri)-r(P)ApoB_S_^Pro^ peptide was obtained from CASLO ApS (Kongens Lyngby, Denmark).

### 2.3. Bacterial Strains and Growth Conditions

Four bacterial strains were used in the present study, i.e., methicillin-resistant *Staphylococcus aureus* (MRSA WKZ-2), *Staphylococcus aureus* ATCC 29213, *Escherichia coli* ATCC 25922, and *Pseudomonas aeruginosa* ATCC 27853. All bacterial strains were grown in Mueller Hinton broth (MHB; Becton Dickinson Difco, Franklin Lakes, NJ, USA) and on tryptic soy agar (TSA; Oxoid Ltd., Hampshire, UK). In all the experiments, bacteria were inoculated and grown over-night in MHB at 37 °C. After 24 h, bacteria were transferred to a fresh MHB tube and grown to mid-logarithmic phase.

### 2.4. Antimicrobial Activity

The antimicrobial activity of (ri)-(r)ApoB_S_^Pro^ peptide was assayed on methicillin-resistant *S. aureus* (MRSA WKZ-2), *S. aureus* ATCC 29213, *E. coli* ATCC 25922, and *P. aeruginosa* ATCC 27853 by using broth microdilution method. Bacteria were grown to the mid-logarithmic phase in MHB at 37 °C and then diluted to 4 × 10^6^ CFU/mL in Difco 0.5X nutrient broth (NB; Becton-Dickenson, Franklin Lakes, NJ, USA). To perform the assay, bacterial samples were mixed 1:1 *v*/*v* with two-fold serial dilutions of the peptidomimetic (0–40 µmol L^−1^) and incubated for 20 h at 37 °C. Following the incubation, each sample was diluted and plated on TSA, in order to count the number of colonies [24,25,26]. All the experiments were carried out as triplicates.

### 2.5. Anti-Biofilm Activity Assays

Anti-biofilm activity assays were performed on methicillin-resistant *S. aureus* (MRSA WKZ-2) and *E. coli* ATCC 25922. Bacterial inocula were grown overnight at 37 °C, then diluted to 4 × 10^9^ CFU/mL in 0.5X MHB medium and mixed 1:1 *v*/*v* with increasing concentrations of the peptidomimetic (0–40 µmol L^−1^). The samples were incubated at 37 °C either for 4 h, in order to test the effects on cell attachment, or for 24 h, in order to assay peptide effects on biofilm formation, as previously described [11,17]. In the case of the crystal violet assay, the bacterial biofilm was washed with phosphate buffer (PBS 1X) and then incubated with the dye (0.04%) for 20 min at room temperature. Following the incubation, samples were washed with PBS and then the dye bound to cells was dissolved in 33% acetic acid. Spectrophotometric measurements were then carried out at a wavelength of 600 nm. Confocal laser scanning microscopy (CLSM) analyses in static conditions were carried out by using Thermo Scientific™ Nunc™ Lab-Tek™ Chambered Coverglass systems (Thermo Fisher Scientific, Waltham, MA, USA). The viability of the cells within the biofilm structure was evaluated by sample staining with a LIVE/DEAD^®^ Bacterial Viability kit (Molecular Probes Thermo Fisher Scientific, Waltham, MA, USA). Staining was performed accordingly to manufacturer’s instructions. Biofilm images were collected by using a confocal laser scanning microscope (Zeiss LSM 710, Zeiss, Germany) and a 63X objective oil-immersion system. Biofilm architecture was analyzed by using the Zen Lite 2.3 software package. Each experiment was performed in triplicate, and all the images were taken under identical conditions.

### 2.6. Circular Dichroism (CD) Spectroscopy Analyses

CD experiments were performed by using a Jasco J-815 circular dichroism spectropolarimeter. The cell path length was 0.1 or 1 cm, depending on the peptide concentration. CD spectra were collected at 25 °C in the far-UV region (190–260 nm) at 0.2 nm intervals, with a 50 nm/min scan rate, 1.0 nm bandwidth, and a 4 s response. Either 4 or 16 accumulations were performed during titration experiments. Each spectrum was corrected by subtracting the background spectrum and reported with a fast-Fourier-transform (FFT) filter. The lyophilized peptidomimetic was dissolved in ultra-pure water (Romil, Waterbeach, Cambridge, GB, UK) at a concentration of 1600 µmol L^−1^, determined on the basis of peptide dry weight and by the bicinchoninic acid (BCA) colorimetric assay (Thermo Fisher Scientific, Waltham, MA, USA). CD spectra of the peptidomimetic were collected in buffer alone (2.5 mM phosphate buffer, pH 7.4) or in the presence of 50% *v*/*v* trifluoroethanol (TFE, Sigma-Merck, Milan, Italy), lipopolysaccharide (LPS) from *E. coli* 0111:B4 strain (Sigma-Merck, Milan, Italy), or lipoteichoic acid (LTA) from *S. aureus* (Sigma-Merck, Milan, Italy). Inverse titration experiments were performed by adding known amounts of peptide in either 0.2 mg/mL LPS or LTA. After addition, samples were mixed with a magnetic stirrer for 3 min prior to CD analyses. CD spectra were corrected by subtracting every time the contribution of the compound under test at any given concentration. CD spectra deconvolutions were performed by using a Microsoft Excel-ported version of the PEPFIT program that is based on peptide-derived reference spectra, in order to estimate secondary structure contents after specular inversion of the raw data [11,27].

### 2.7. Preparation of HA-BDDE Hydrogel Loaded with (ri)-r(P)ApoB_S_^Pro^ Peptidomimetic

Peptidomimetic (ri)-r(P)ApoB_S_^Pro^ was added to hyaluronic acid hydrogel cross-linked with 2.5% 1,4-butanediol diglycidyl ether (HA-BDDE) at a ratio of 4:1 (*v*/*v*). A solution of peptidomimetic at a concentration of 80 or 320 μmol L^−1^ was mixed with HA-BDDE (density of 1 g mL^−1^), properly stirred, and then lyophilized for 24 h, in order to obtain white spongy samples [28,29]. Control samples were prepared under the same experimental conditions but in the absence of the peptidomimetic. Prior to all the characterizations, dried hydrogels were rehydrated and sterilized by exposure to UV light for 20 min. 

### 2.8. Eukaryotic Cell Culture and Biocompatibility Evaluation of (ri)-r(P)ApoB_S_^Pro^ and of HA-BDDE Hydrogel System

Immortalized human keratinocytes (HaCaTs) and human dermal fibroblasts (HDFs) were cultured in high-glucose Dulbecco’s modified Eagle’s medium (DMEM) supplemented with 10% fetal bovine serum (FBS), 1% antibiotics (Pen/strep), and 1% L-glutamine and grown at 37 °C in a humidified atmosphere containing 5% CO_2_. To evaluate peptidomimetic biocompatibility, cells were seeded into 96-well plates at a density of 3 × 10^3^ cells/well in 100 µL of complete DMEM for 24 h prior to the treatment. Cells were then incubated in the presence of increasing peptidomimetic concentrations (0–20 µmol L^−1^) for 24, 48, and 72 h. When evaluating the biocompatibility of the HA-BDDE hydrogel system, cells were seeded into 96-well plates at a density of 6 × 10^3^ cells/well in 100 µL of complete DMEM for 24 h prior to the treatment. Following incubation, cells were treated with HA-BDDE hydrogel hydrated with growth medium and previously loaded with the peptidomimetic at a final concentration of 80 or 320 μmol L^−1^. In all cases, following treatment, cell culture supernatants were replaced with 0.5 mg/mL MTT (3-(4,5-dimethylthiazol-2-yl)-2,5-diphenyltetrazolium bromide) reagent dissolved in DMEM medium without red phenol (100 µL/well). After 4 h of incubation at 37 °C, the formazan salts were solubilized in 0.01 M HCl in anhydrous isopropanol, and the absorbance of the obtained samples was measured at λ = 570 nm using an automatic plate reader spectrophotometer (Synergy™ H4 Hybrid Microplate Reader, BioTek Instruments, Inc., Winooski, VT, USA), as previously described [25]. Cell survival was expressed as the mean of the percentage values compared to control untreated cells. 

### 2.9. Hemolytic Activity

The release of hemoglobin from human erythrocytes was used as a measure for the hemolytic activity of (ri)-r(P)ApoB_S_^Pro^. Briefly, human red blood cells (RBCs) were collected from EDTA anti-coagulated blood, washed three times by centrifugation at 800× *g* for 10 min, and 200-fold diluted in PBS pH 7.4. Aliquots of diluted erythrocytes (75 μL) were added to the peptide solution (0–40 μmol L^−1^; 75 μL) in 96-well microtiter plates, and the mixtures were incubated for 1 h at room temperature. Following the incubation, the plate was centrifuged for 10 min at 1300× *g*, and 100 μL of supernatant from each well was transferred to a new 96-well plate, as previously described [25]. Absorbance values were determined at 405 nm by using an automatic plate reader (Synergy™ H4 Hybrid Microplate Reader, BioTek Instruments, Inc., Winooski, VT, USA). The percentage of hemolysis was determined by comparison with the control samples containing PBS (negative control) or 1% (*v*/*v*) SDS in PBS solution (positive control, complete lysis).

(1) $$\mathrm{Hemolysis}\;(\%)=\frac{\left(\mathrm{Abs}405\;\mathrm{nm}\;\mathrm{peptide}\;-\;\mathrm{Abs}405\;\mathrm{nm}\;\mathrm{negative}\;\mathrm{control}\right)}{\left(\mathrm{Abs}405\;\mathrm{nm}\;\mathrm{positive}\;\mathrm{control}\;-\;\mathrm{Abs}405\;\mathrm{nm}\;\mathrm{negative}\;\mathrm{control}\right)}\times 100$$

### 2.10. Cell Infection Assay

Immortalized human keratinocytes (HaCaTs) were seeded into 24-well plates at a density of 3 × 10^5^ cells/well and allowed to attach for 24 h at 37 °C in a humidified atmosphere containing 5% CO_2_. Following incubation, cells were washed three times with PBS and infected with *E. coli* ATCC 25922 at multiplicity of infection (MOI) of 2 in the presence or absence of (ri)-r(P)ApoB_S_^Pro^ 10 μmol L^−1^. To measure the number of bacteria in the wells, the keratinocytes were washed three times with PBS, and 500 µL of 1% Triton X-100 was added in order to detach and lyse the cells. Samples containing the detached keratinocytes were serially diluted, plated onto TSA agar plates, and incubated overnight at 37 °C to count the number of colonies. The number of bacteria present in the samples was analyzed over time (0–2–4 h).

### 2.11. Characterization of Swelling Properties of HA-BDDE Hydrogel Loaded with (ri)-r(P)ApoB_S_^Pro^ Peptidomimetic

The hydrogel’s swelling properties were characterized by measuring the gravimetric change over time as described by Jie Zhu et al. [30]. Briefly, the freeze-dried hydrogels were weighed (*S*_0_) and then immersed into distilled water. To obtain the swelling ratio, hydrogels samples were weighed (*S*_1_) at different time intervals until swelling equilibrium was reached. Each sample was measured in three replicates. The hydrogel’s swelling ratio was calculated as follows:

(2) $$\mathrm{Swelling}\;\mathrm{ratio}\;(\%)=\frac{S_{1}-S_{0}}{S_{0}}\times 100$$

### 2.12. Degradation Analyses of HA-BDDE Hydrogel Loaded with (ri)-r(P)ApoB_S_^Pro^ Peptidomimetic

The swollen hydrogels were weighed (*W*_0_), and degradation performances were evaluated by incubating the samples with 300 U mL^−1^ of hyaluronidase (ref. H3506, Sigma-Merck) solubilized in 0.02 M sodium phosphate, pH 7.4, containing 0.01% bovine serum albumin and 77 mM NaCl, as described by Wanxu Cao et al. [31]. The mixtures were placed in a water bath at 37 °C, and the weight loss percentage was calculated by weighing samples (*W*_1_) at defined time intervals. Every sample was measured in three replicates. Degradation behavior was expressed as the percentage of weight loss and was calculated as follows:

(3) $$\mathrm{Weight}\;\mathrm{change}\;(\%)=\frac{W_{1}}{W_{0}}\times 100$$

### 2.13. Rheological Characterization of HA-BDDE Hydrogel Loaded with (ri)-r(P)ApoB_S_^Pro^ Peptidomimetic

Rheological measurements were performed by using an Anton Paar MCR302 rheometer with d = 50 mm cone geometry (cone angle 1°, gap 101 μm). Briefly, 2 mL of fresh HA-BDDE hydrogel or HA-BDDE hydrogel loaded with 320 μmol L^−1^ of (ri)-r(P)ApoB_S_^Pro^ were deposited between the cone and the plate, and the parameters (G’—storage modulus, and G”—loss modulus) were followed at 37 °C at a constant frequency of 1.59 Hz.

### 2.14. In Vitro Peptidomimetic Release from Hydrogel System

To determine peptidomimetic release, HA-BDDE hydrogels loaded with 80 or 320 μmol L^−1^ (ri)-r(P)ApoB_S_^Pro^ were immersed in distilled water or saline buffer (30 mM HEPES buffer + 50 mM NaCl to reach pH 7 and 30 mM CH_3_COOH buffer + 50 mM NaCl to reach pH 5), in order to simulate the skin ionic strength according to Traeger and co-workers [32], and then incubated at 37 °C over time. At defined time points, aliquots of 100 μL were collected and 100 μL of water were added, in order to keep a constant volume. The amounts of released (ri)-r(P)ApoB_S_^Pro^ were measured by HPLC analyses performed by using a Shimadzu LC-20 Prominence (Shimadzu Corporation, Kyoto, Japan) mounted with a thermostated PDA detector (SPD-M20A). For this purpose, samples were loaded on a Phenomenex Aeris Peptide XB-C18 3.6 µm column (150 × 4.6 mm), and eluted with a linear gradient of ultra-pure water (A) and acetonitrile (B) (UpS solvent, Romil) from 15% to 95% of solvent B over 20 min. TFA (0.1% *v*/*v*) was added to both solvents. To quantify the released peptidomimetic, a five-point calibration curve was used. Each calibration standard solution was prepared by diluting the primary standard (1600 µmol L^−1^ determined on the basis of peptidomimetic dry weight) in the working buffer (ultra-pure water), in order to obtain the following concentrations: 2.5, 5, 10, 20, and 40 µmol L^−1^. Linear regression was used to fit the peak areas as integrated from the 220 nm chromatogram (R^2^ = 0.997). A blank sample was included in each calibration curve. Two separate analyses of peptidomimetic release were performed on a blind and randomized basis, by injecting all the samples collected at different time intervals (0, 0.5, 1, 2, 3, 24, 48, 72 h). For quality control, blank, zero, and spiked samples (5, 10, and 40 µmol L^−1^) were injected, with the latter being found within 10% deviation of the nominal concentration.

### 2.15. Antimicrobial Activity of HA-BDDE Hydrogel System Loaded with the Peptidomimetic

The antimicrobial activity of HA-BDDE hydrogel loaded with (ri)-r(P)ApoB_S_^Pro^ was assayed on methicillin-resistant *S. aureus* (MRSA WKZ-2) and *E. coli* ATCC 25922 bacterial strains. Bacteria were grown to mid-logarithmic phase in MHB at 37 °C. Afterward, cells were diluted to 4 × 10^6^ CFU/mL in Difco 0.5X NB (Becton-Dickenson, Franklin Lakes, NJ, USA) and mixed 1:1 *v*/*v* with HA-BDDE hydrogel loaded with the peptidomimetic at a final concentration of 80 or 320 μmol L^−1^. Following overnight incubation, each sample was diluted, plated on TSA, and incubated at 37 °C for 24 h, in order to count the number of colonies. All the experiments were carried out as triplicates.

### 2.16. Migration Assay

To examine whether the hydrogel system loaded with the peptidomimetic is able to counteract bacterial migration across surfaces, the upper chambers of a trans-well plate (Costar 3422^®^, Corning Corporation, Corning, NY, USA) were coated with control saline solution, HA-BDDE hydrogel or HA-BDDE hydrogel loaded with (ri)-r(P)ApoB_S_^Pro^, as described by Xiaojuan Li et al. [33]. Following coating, methicillin-resistant *S. aureus* (MRSA WKZ-2) and *E. coli* ATCC 25922 bacterial cells were diluted to 4 × 10^6^ CFU/mL in Difco 0.5X NB (Becton-Dickinson, Franklin Lakes, NJ, USA), and plated into the previously coated upper chambers. The medium in the lower wells was then analyzed at defined time intervals, in order to monitor bacterial cells’ migration from the upper to the lower wells. The number of migrated bacteria was quantified by diluting and plating each sample on TSA. Following an incubation of 24 h at 37 °C, the number of colonies was counted. The experiment was performed in three independent replicates. 

### 2.17. Scanning Electron Microscopy Analyses of Bacterial Cells Treated with HA-BDDE Hydrogel System Loaded with the Peptidomimetic

To perform scanning electron microscopy (SEM) analyses, methicillin-resistant *S. aureus* (MRSA WKZ-2) and *E. coli* ATCC 25922 cells at a density of 4 × 10^6^ CFU/mL in Difco 0.5X NB were mixed 1:1 *v*/*v* with HA-BDDE hydrogel loaded with the peptidomimetic at a final concentration of 80 or 320 μmol L^−1^. Following overnight incubation at 37 °C, untreated methicillin-resistant *S. aureus* (MRSA WKZ-2) cells were centrifuged at 10,000 rpm at 4 °C, since a complete hydrolysis of hydrogel matrix due to bacteria was observed. In the case of peptide-treated methicillin-resistant *S. aureus* (MRSA WKZ-2) cells and all *E. coli* ATCC 25922 samples, mixtures were, instead, directly placed on a glass slide and then fixed in 2.5% glutaraldehyde at the end of the incubation. After 20 h incubation, the samples were washed three times in distilled water (dH_2_O) and dehydrated with a graded ethanol series: 25% ethanol (1 × 10 min); 50% ethanol (1 × 10 min); 75% ethanol (1 × 10 min); 95% ethanol (1 × 10 min); 100% anhydrous ethanol (3 × 30 min). At the end of the alcoholic dehydration process, methicillin-resistant *S. aureus* (MRSA WKZ-2) samples were also deposited into the glass substrate, and all the samples were sputter-coated with a thin layer of Au-Pd (Sputter Coater Denton Vacuum Desk V) to allow their subsequent morphological characterization using a FEI Nova NanoSEM 450 at an accelerating voltage of 5 kV with an Everhart Thornley Detector (ETD) and a Through Lens Detector (TLD) at high magnification.

## 3. Results

### 3.1. In Vitro Antimicrobial and Anti-Biofilm Activity of Synthetic Retro-Inverso r(P)ApoB_S_^Pro^

**Figure 1:**
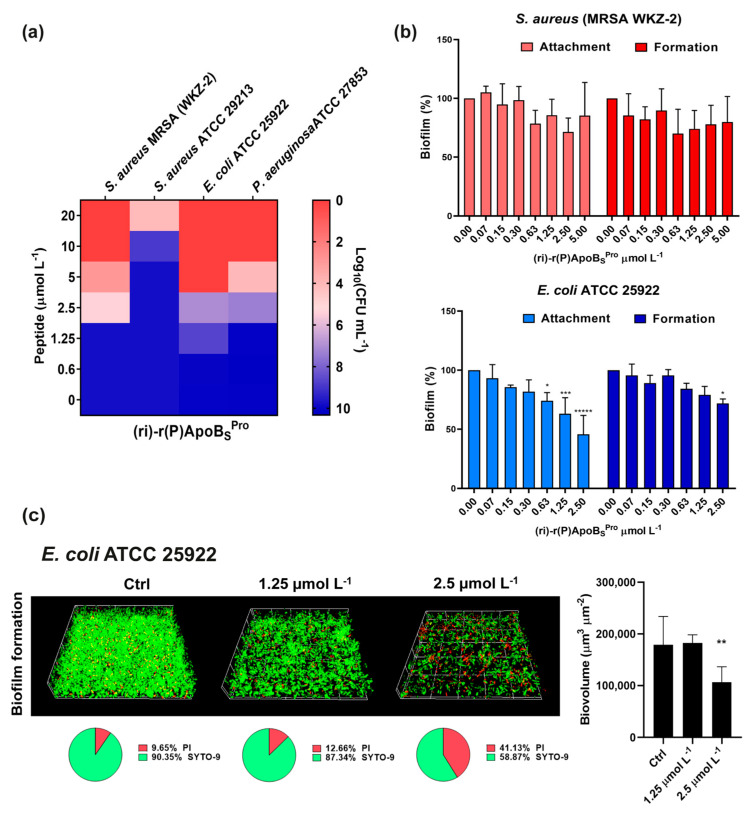
Antimicrobial and anti-biofilm activities of retro-inverso synthetic peptide. (**a**) Antimicrobial activity of (ri)-r(P)ApoB_S_^Pro^ (μmol L^−1^) against four bacterial strains; reported data refer to assays performed in triplicate and heat maps show averaged log_10_(CFU mL^−1^) values. (**b**) Anti-biofilm activity of (ri)-r(P)ApoB_S_^Pro^ on methicillin-resistant *S. aureus* (MRSA WKZ-2) and *E. coli* ATCC 25922 strains. The effects of increasing concentrations of (ri)-r(P)ApoB_S_^Pro^ peptide were evaluated on cells attachment and biofilm formation. Data represent the mean (±standard deviation, SD) of at least three independent experiments, each one carried out with triplicate determinations. (**c**) Effects of (ri)-r(P)ApoB_S_^Pro^ on biofilm formation in the case of *E. coli* ATCC 25922 by CLSM imaging. Two-dimensional structures of the biofilms were obtained by confocal z-stack using Zen Lite 2.3 software. Biovolume (µm^3^ µm^−2^) values were calculated by using Zen Lite 2.3 software. Significant differences were indicated as * *p* < 0.05, ** *p* < 0.01, *** *p* < 0.001, or ***** *p* < 0.00001 for treated vs. control samples, each experiment was carried out in triplicate.

Firstly, we assessed the antimicrobial activity of the peptidomimetic (ri)-r(P)ApoB_S_^Pro^ against a panel of four Gram-negative and Gram-positive bacterial strains, selected among those more frequently isolated from skin and soft tissue infections [34,35,36]. The peptidomimetic was found to inhibit the bacterial growth at concentration values ranging from 5 to 10 μmol L^−1^ in the case of *P. aeruginosa* ATCC 27853 and *E. coli* ATCC 25922, and at concentration values ranging from 10 to 20 μmol L^−1^ in the case of *S. aureus* ATCC 29213 and methicillin-resistant *S. aureus* (MRSA WKZ-2). Based on these results, we can conclude that the peptidomimetic is endowed with significant broad-spectrum antimicrobial properties (Figure 1a). Furthermore, (ri)-r(P)ApoB_S_^Pro^ was found to inhibit (40–50%) biofilm adhesion and formation when tested on Gram-negative *E. coli* ATCC 25922 (Figure 1b). No significant anti-biofilm effects were detected, instead, when the peptide was tested on Gram-positive methicillin-resistant *S. aureus* (MRSA WKZ-2) by performing crystal violet assays (Figure 1b). To further investigate the peptidomimetic anti-biofilm activity on *E. coli* ATCC 25922 strain, confocal laser scanning microscopy (CLSM) analyses were also performed, and it was demonstrated that (ri)-r(P)ApoB_S_^Pro^ at a concentration of 2.5 μmol L^−1^ is able to significantly affect the biofilm formation of *E. coli* ATCC 25922 by determining a significant reduction of biofilm biovolume (Figure 1c). Such results appear to be in agreement with the findings previously obtained for natural ApoB-derived antimicrobial peptides, whose antimicrobial properties strictly depend on specific features of the bacterial strains under test [8].

### 3.2. Conformational Analyses of (ri)-r(P)ApoB_S_^Pro^ Peptide by Far-UV Circular Dichroism 

**Figure 2:**
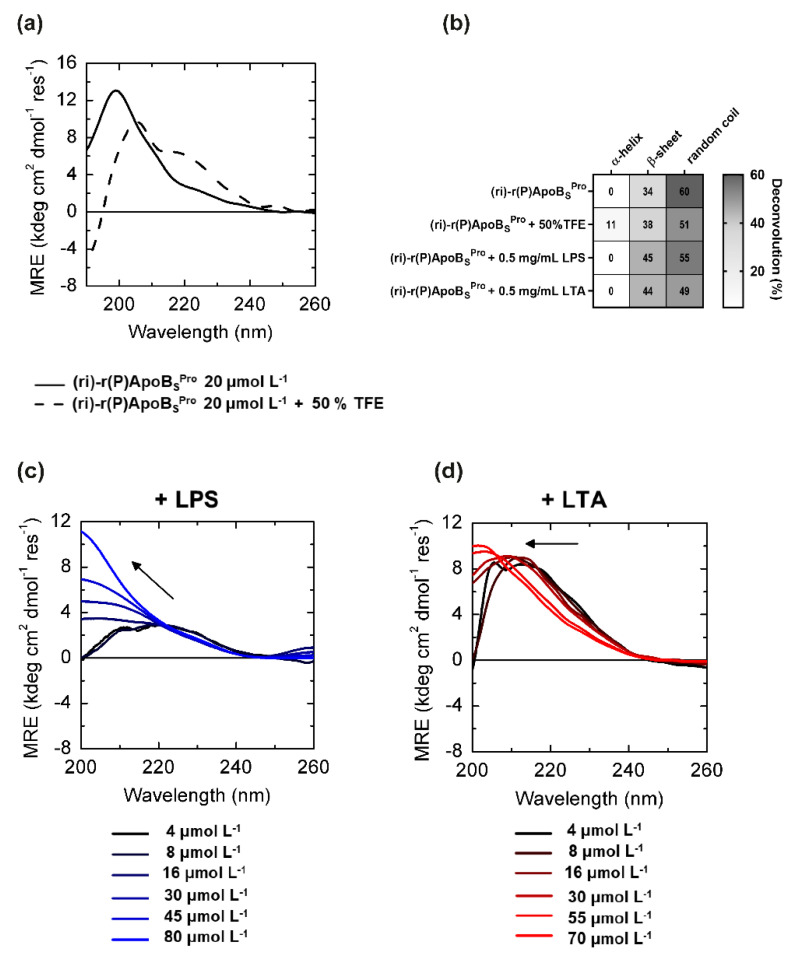
Conformational analyses of (ri)-r(P)ApoB_S_^Pro^ peptide by far-UV circular dichroism. (**a**) CD spectra of peptidomimetic (ri)-r(P)ApoB_S_^Pro^ 20 μmol L^−1^ in the absence (black line) or in the presence (black dashed line) of 50% (*v*/*v*) TFE. (**b**) CD spectra deconvolution percentages of (ri)-r(P)ApoB_S_^Pro^ peptide in 2.5 mM sodium phosphate pH 7.4 buffer and in the presence of 50% TFE, 0.5 mg mL^−1^ LPS or LTA. Secondary structure percentages were calculated using CDPRO software. CD spectra at different concentrations of (ri)-r(P)ApoB_S_^Pro^ in the presence of constant amounts (0.2 mg mL^−1^) of either LPS (**c**) or LTA (**d**).

Far-UV CD spectroscopy was used to analyze the correlation between peptidomimetic antimicrobial activity and conformations. The retro-inverso peptide appeared largely unstructured in aqueous buffer (2.5 mM phosphate buffer, pH 7.4), as evidenced by the broad maximum centered at 199 nm in the CD spectra (Figure 2a). However, it should be highlighted that the molar residue ellipticity appeared significantly lower in absolute values with respect to that previously obtained for its (L)-amino acid variant [11] (~13 kdeg cm^2^ dmol^−1^ res^−1^ vs. ~30 kdeg cm^2^ dmol^−1^ res^−1^ for (ri)-r(P)ApoB_S_^Pro^ and r(P)ApoB_S_^Pro^, respectively), thus suggesting the presence of a higher content of secondary structure elements in the case of the peptidomimetic. Indeed, deconvolution analyses revealed the presence of approximately 34% of β-sheet secondary structure content, whereas previously characterized r(P)ApoB_S_^Pro^ was found to assume a fully random coil conformation [11]. When we analyzed the effects of 50% TFE, a widely adopted secondary structure inducer, (ri)-r(P)ApoB_S_^Pro^ assumed a partial (~10%) helical conformation (Figure 2a), as evidenced by the presence of two broad minima at around 208 and 222 nm, and a maximum at <200 nm. This behavior is similar to that previously described for its (L)-amino acid variant r(P)ApoB_S_^Pro^ peptide [11].

More interestingly, differently from its (L)-peptide counterpart [11], the retro-inverso variant’s secondary structure was significantly altered upon incubation with lipopolysaccharide (LPS), the predominant glycolipid in the outer membrane of Gram-negative bacteria. When increasing amounts of peptidomimetic were incubated with 0.2 mg/mL LPS endotoxin, the maximum wavelength progressively shifted from 220 to 200 nm (Figure 2c), suggesting a major switch toward a β-strand conformation at higher LPS:peptide ratios, probably due to an interaction between the peptidomimetic and LPS. Considering that the peptidomimetic exerts a stronger direct antimicrobial activity on Gram-negative bacteria as opposed to Gram-positive bacteria, we hypothesized a less pronounced effect induced on peptide conformation by lipoteichoic acid (LTA), an anionic glycerol phosphate polymer of the cell wall of Gram-positive strains. Indeed, when increasing amounts of the peptidomimetic were incubated with 0.2 mg/mL LTA from *S. aureus*, less-pronounced variations of the CD spectrum were observed (Figure 2d). In particular, band maximum wavelength shifted only from 215 to 200 nm with a far lower effect on the molar residue ellipticity, most probably due to only minimal β-strand formation. CD spectra deconvolutions are summarized in Figure 2b. Data appear in agreement with the higher antimicrobial activity of the peptidomimetic (ri)-r(P)ApoB_S_^Pro^ against Gram-negative bacteria compared to Gram-positive strains. Such results appear even more interesting considering the recently published observations indicating that the antimicrobial activity of two natural versions of the antimicrobial peptide identified in human ApoB depends on their interaction with specific components of bacterial surfaces, such as LPS or LTA, which induces peptides to form β-sheet-rich amyloid-like structures that are probably responsible for their antibacterial activity [15].

### 3.3. Biocompatibility of (ri)-r(P)ApoB_S_^Pro^ toward Human Skin Cells

**Figure 3:**
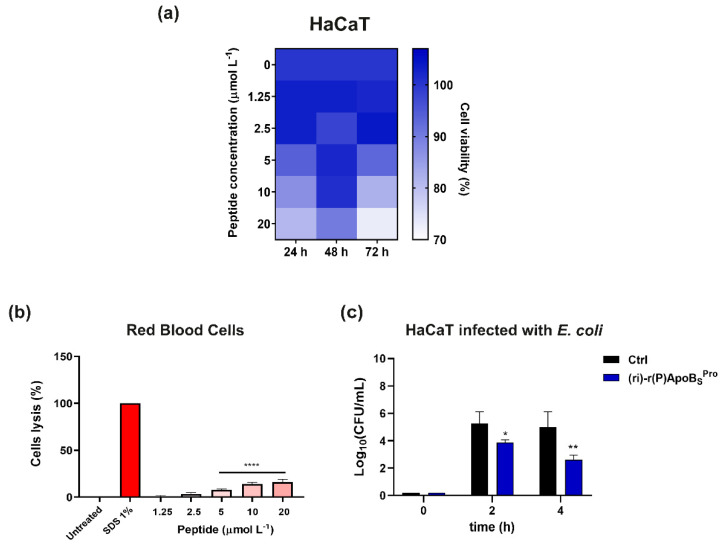
Effects of retro-inverso synthetic peptide on human eukaryotic cell lines. (**a**) Cytotoxic effects of increasing concentrations of (ri)-r(P)ApoB_S_^Pro^ on HaCaT (immortalized human keratinocytes) cells. Cell viability was assessed by MTT assays and expressed as the percentage of viable cells compared to untreated cells (control). (**b**) Analysis of hemolytic effects of (ri)-r(P)ApoB_S_^Pro^ toward human red blood cells (RBCs) after 1 h of incubation at 37 °C. Data represent the mean (±standard deviation, SD) of at least three independent experiments, each one carried out with triplicate determinations. (**c**) Effects of (ri)-r(P)ApoB_S_^Pro^ peptide on the adhesion of *E. coli* bacterial cells to HaCaT cells. Experiments were performed three times in duplicate. Error bars represent the standard deviation of the mean. Significant differences were indicated as * *p* < 0.05, ** *p* < 0.01 or **** *p* < 0.0001 for treated vs. control samples.

We performed additional experiments to verify the biocompatibility of (ri)-r(P)ApoB_S_^Pro^ toward skin cell cultures and to exclude hemolytic effects that may result from incorporation of D-amino acids into peptide sequences [37]. Peptidomimetic (ri)-r(P)ApoB_S_^Pro^ did not significantly affect cell viability, as indicated by the MTT assay results (Figure 3a). Indeed, the viability of immortalized human keratinocytes (HaCaTs) was found to be unchanged upon exposure to increasing concentrations of (ri)-r(P)ApoB_S_^Pro^ for different time intervals (Figure 3a). Moreover, when we tested (ri)-r(P)ApoB_S_^Pro^ on human red blood cells (RBCs), slight lytic effects were observed only at the highest peptidomimetic concentrations tested, thus indicating that the peptidomimetic is endowed with a good profile of biocompatibility (Figure 3b).

Since skin and soft tissue infections occur when bacteria adhere to host cells, the ability of the peptidomimetic to prevent the adhesion of pathogens to skin cells was also evaluated. For this purpose, human keratinocytes infected with *E. coli* ATCC 25922 cells were treated with (ri)-r(P)ApoB_S_^Pro^. After a 4 h incubation, the synthetic retro-inverso peptide was able to reduce by 50% the number of bacterial cells infecting keratinocytes with respect to control untreated cells (Figure 3c). This property opens interesting perspectives to the future applicability of (ri)-r(P)ApoB_S_^Pro^ in the development of therapeutic strategies to treat skin infections.

### 3.4. Swelling and Degradation Profiles of HA Hydrogel System Loaded with (ri)-r(P)ApoB_S_^Pro^

**Figure 4:**
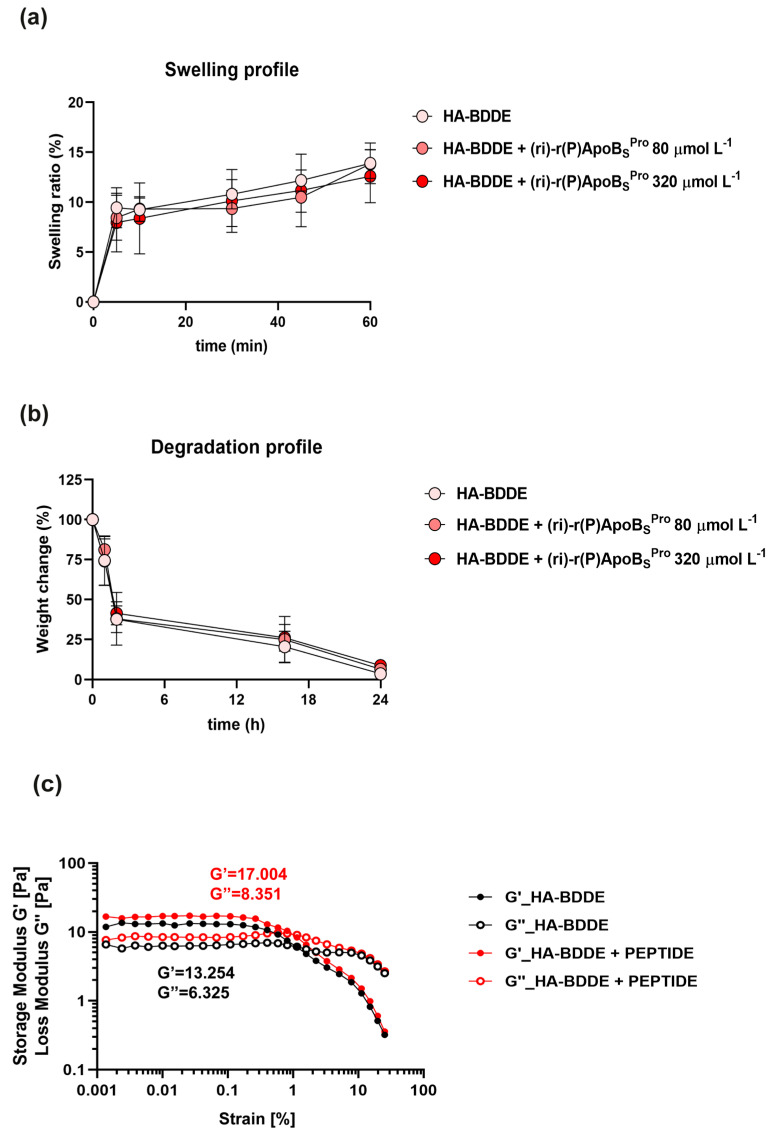
Physical and mechanical properties of the hydrogel-based system. Swelling (**a**) and degradation (**b**) profiles of HA-BDDE alone and loaded with two different concentrations of (ri)-r(P)ApoB_S_^Pro^ (80 or 320 μmol L^−1^). (**c**) Rheological characterization of HA-BDDE hydrogel alone and loaded with (ri)-r(P)ApoB_S_^Pro^ peptidomimetic (320 μmol L^−1^) based on the monitoring of elastic (G’) and viscous moduli (G”) as a function of shear stress. The values reported, 17.004 and 8.351, and 13.254 and 6.326 for G’ and G”, respectively, are the parameters obtained for each sample in the linear viscous range.

To identify a suitable system to topically deliver the peptidomimetic, a proper HA-BDDE hydrogel system loaded with (ri)-r(P)ApoB_S_^Pro^ was prepared as described in Section 2.7 of Section 2. The ability to retain a high amount of water is one of the key properties of hydrogel-based systems. Indeed, they can swell in water under physiological conditions without dissolving. The swelling capability plays a key role in keeping the injured site moist and in controlling bleeding. Moreover, this mechanical characteristic is fundamental to allow molecules to diffuse into or to be released from the hydrogels [30]. The swelling properties of hyaluronic acid gels can be affected by pH, ionic strength, temperature, and composition of the surrounding solution [38]. We examined whether the presence of the peptidomimetic could affect swelling and degradation profiles of HA-BDDE. As shown in Figure 4a, HA-BDDE hydrogels loaded with two different concentrations of the peptidomimetic (80 or 320 μmol L^−1^) retained a swelling behavior similar to that of control sample, thus indicating that the presence of the peptidomimetic does not affect the ability of the system to absorb water and to expand. Similarly, the degradation profile was found to be unaffected by the presence of the peptide, since the activity of hyaluronidase enzyme, which acts by cleaving the (1→4)-linkages between N-acetylglucosamine and glucuronate, was found to be unmodified in the presence of the peptidomimetic (Figure 4b). Hence, unlike covalently functionalized peptides, which significantly affect the swelling capacity of hydrogels, (ri)-r(P)ApoB_S_^Pro^ encapsulated at high concentrations does not alter the mechanical properties of the hydrogel [39]. Altogether, these findings point to the proposed system as an effective approach to deliver the peptidomimetic to the site of infection.

### 3.5. Rheological Analyses of HA Hydrogel System Loaded with (ri)-r(P)ApoB_S_^Pro^

To analyze the rheological properties of the developed hydrogel-based systems, we evaluated the elastic modulus G’ (storage modulus) and the viscous modulus G” (loss modulus) in amplitude, and we determined those parameters in the range of the linear viscoelastic region (LVR) for each sample (Figure 4c). The HA-BDDE hydrogel system loaded with (ri)-r(P)ApoB_S_^Pro^ showed values of G’ and G” (17.004 and 8.351, respectively) slightly higher than the control (13.254 and 6.326, respectively), thus indicating that the presence of the peptidomimetic within the gel might increase its viscosity by establishing weak links among the HA-filaments. However, the tan(δ) parameters of HA-BDDE hydrogel alone or loaded with the peptidomimetic are comparable (G”/G’ = 0.47 and G”/G’ = 0.49, respectively), thus demonstrating that (ri)-r(P)ApoB_S_^Pro^ does not alter the “gel-like” structure [40] of the system (Figure 4c). This is probably due to the non-covalent bonds between the hydrogel chains and the peptide molecules that do not significantly affect the viscosity parameters as instead demonstrated for peptides that are covalently bound to the hydrogel polymers [41]. This represents an important finding, underlining that the presence of the peptidomimetic does not influence the main features that make hydrogel-based systems an ideal tool to topically deliver therapeutic agents.

### 3.6. Peptide Release from the Hydrogel System

**Figure 5:**
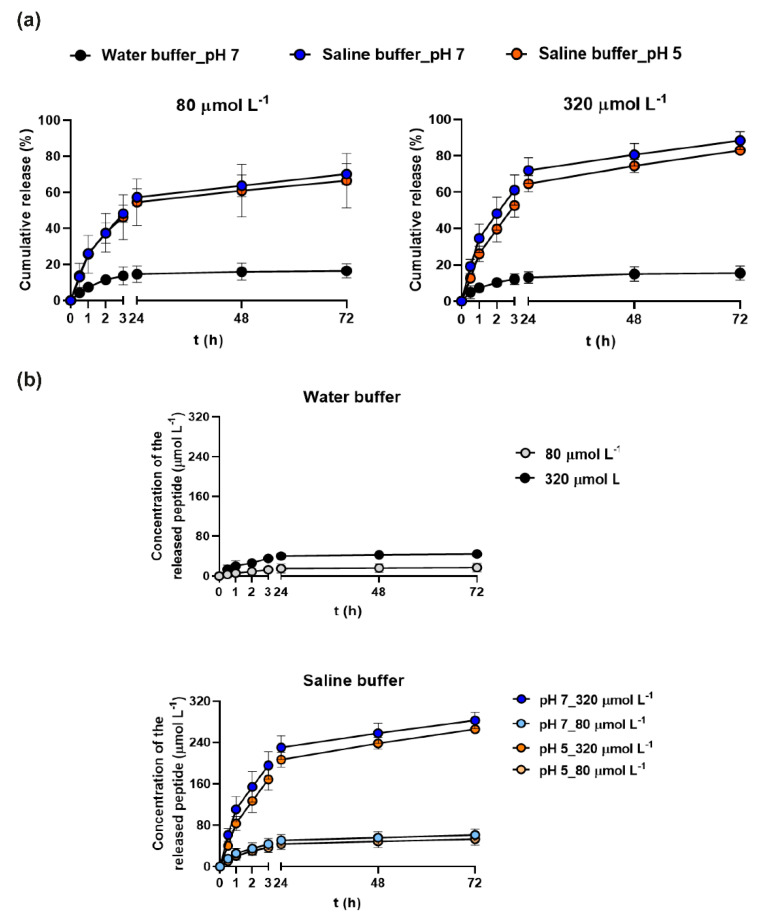
In vitro peptidomimetic release profile. (**a**) Evaluation of the amount of the peptidomimetic released from HA-BDDE loaded with two different (ri)-r(P)ApoB_S_^Pro^ concentrations, i.e., 80 or 320 μmol L^−1^. (**b**) Cumulative release of (ri)-r(P)ApoB_S_^Pro^ expressed as percentage with respect to the total initial amount of peptidomimetic filled into gel.

Since a controlled and gradual release of the therapeutic agent is essential for the construction of a suitable drug delivery system, we performed experiments aimed at evaluating whether (ri)-r(P)ApoB_S_^Pro^ is released over time, upon loading into the hydrogel. For this purpose, the HA-BDDE hydrogel system loaded with 80 or 320 μmol L^−1^ (ri)-r(P)ApoB_S_^Pro^ was immersed in water or in saline buffer at pH 7 or 5, in order to simulate the pH of skin barrier in normal and pathological conditions, respectively [42]. The ionic strength of the saline buffer was designed to simulate that of sweat [32]. To evaluate peptidomimetic release, aliquots were collected at defined time intervals, in order to estimate the amount of the released peptidomimetic by HPLC analyses. Results indicate that the hydrogel was able to release 80% of the peptidomimetic amount when in contact with the saline buffer (independently from pH value) with respect to the water buffer, in which the maximum percentage of the released antimicrobial was 15% (Figure 5a). These data indicate that the selected system is able to release the antimicrobial by responding to external stimuli, such as the ionic strength, mimicking the sweat of the skin barrier. It is also important to notice that the percentage of release of the peptidomimetic always appeared to be proportional to the amount of (ri)-r(P)ApoB_S_^Pro^ initially loaded into the hydrogel (Figure 5b). It has to be highlighted that swellable systems are generally able to release water-soluble molecules upon the entry of water molecules into the gel, swelling of the matrix, and consequent drug diffusion [43]. The behavior here observed for peptide release seems to fit a linear Fickian curve [44], since it is characterized by an initial linear increase followed by the gradual reaching of a plateau (Figure 5).

### 3.7. Antimicrobial Properties of HA-BDDE Loaded with (ri)-r(P)ApoB_S_^Pro^

**Figure 6:**
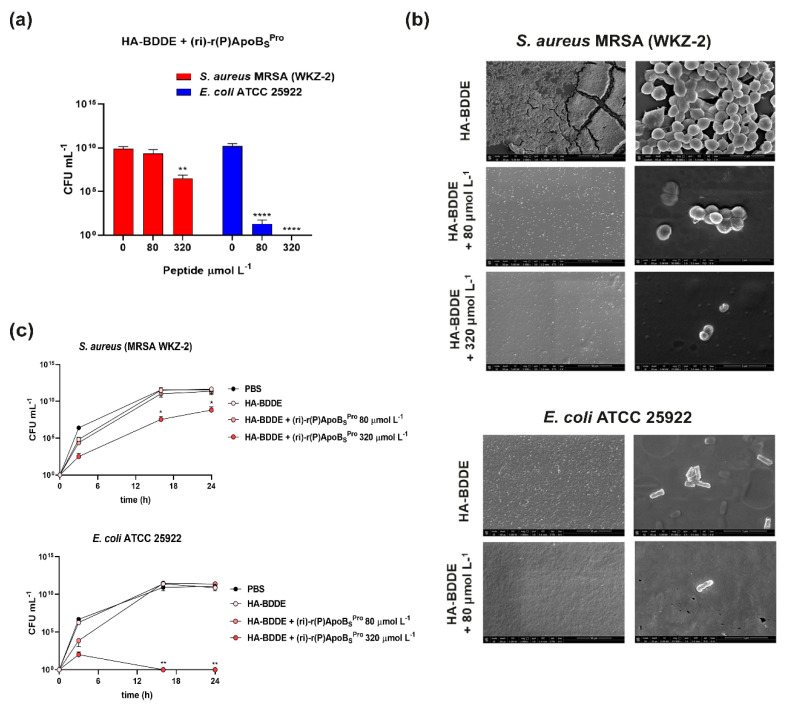
Antimicrobial activity of HA-BDDE hydrogel functionalized with the peptide. (**a**) Antimicrobial efficacy of HA-BDDE hydrogel system loaded with (ri)-r(P)ApoB_S_^Pro^ against methicillin-resistant *S. aureus* (MRSA WKZ-2) and *E. coli* ATCC 25922 bacterial strains; reported data refer to assays performed in triplicate. (**b**) Morphological analyses by SEM of methicillin-resistant *S. aureus* (MRSA WKZ-2) and *E. coli* ATCC 25922 bacterial strains treated with HA-BDDE functionalized or not with r(P)ApoB_S_^Pro^. (**c**) Bacterial migration across surfaces coated with PBS, unfunctionalized HA-BDDE hydrogel system, or HA-BDDE hydrogel system functionalized with (ri)-r(P)ApoB_S_^Pro^. Significant differences were indicated as * *p* < 0.05, ** *p* < 0.0 and **** *p* < 0.0001 for treated vs. control samples.

In order to evaluate whether the newly developed hydrogel-based system loaded with the peptidomimetic guarantees the preservation of the antimicrobial activity of (ri)-r(P)ApoB_S_^Pro^, we performed analyses on methicillin-resistant *S. aureus* (MRSA WKZ-2) and *E. coli* ATCC 25922, as representatives of Gram-positive and Gram-negative bacterial strains, respectively. We determined the minimal inhibitory concentration (MIC) values by counting the number of bacterial colonies obtained after an overnight incubation with HA-BDDE hydrogels loaded with (ri)-r(P)ApoB_S_^Pro^ or unloaded. Significant antibacterial effects were detected against both bacterial strains (Figure 6a). In detail, methicillin-resistant *S. aureus* (MRSA WKZ-2) was susceptible to the hydrogel system loaded with the highest concentration of peptidomimetic (MIC_40_ = 320 μmol L^−1^) (Figure 6a). *E. coli* ATCC 25922 was even more susceptible with an MIC_90_ value corresponding to 80 μmol L^−1^, and a complete growth inhibition (MIC_100_) obtained at a concentration of peptidomimetic of 320 μmol L^−1^ (Figure 6a). These observations are in agreement with previously reported data indicating that (ri)-r(P)ApoB_S_^Pro^ exerts stronger toxic effects on Gram-negative than on Gram-positive bacterial strains (Figure 1a). To further investigate the antimicrobial properties of HA-BDDE hydrogels loaded with (ri)-r(P)ApoB_S_^Pro^, scanning electron microscopy (SEM) analyses were also performed. As shown in Figure 6b, when methicillin-resistant *S. aureus* (MRSA WKZ-2) cells were incubated with the HA-BDDE hydrogel system in the absence of the peptidomimetic, an almost complete degradation of the structure of the HA-hydrogel was observed upon incubation. Indeed, the activity of staphylococcal hyaluronidases has been reported to be a “spreading factor” contributing to the increase of lesion sizes in skin infections [45]. The phenomenon appeared slighter in the presence of the peptidomimetic than in to control samples. This, combined with a dose-dependent inhibition of cell viability observed in the presence of the peptidomimetic, highlights significant anti-infective properties, with the strongest effects observed at the highest peptidomimetic concentration tested (320 μmol L^−1^) (Figure 6b). When the same experiments were performed on *E. coli* ATCC 25922 cells, similar results were obtained with the only exception that an almost complete inhibition of cell growth was already observed in the presence of the HA-BDDE hydrogel system loaded with 80 μmol L^−1^ peptidomimetic (Figure 6b). Indeed, the number of *E. coli* bacterial cells within and surrounding the gel was significantly reduced in the sample treated with the peptidomimetic than in the control. It has to be highlighted that, in the case of the *E. coli* sample, no degradation of the hydrogel matrix was observed, with a consequent spreading of cells on multiple layers and the visualization of a lower number of cells on the upper surface even in the control (Figure 6b).

To investigate the antimicrobial activity of the HA-BDDE hydrogel system loaded with the peptidomimetic more deeply, we also performed experiments to evaluate whether the hydrogel system was able to counteract bacterial migration across surfaces. For this purpose, we performed trans-well migration assays by coating the upper chambers of a trans-well plate with PBS, HA-BDDE hydrogel system in the absence of the peptide, or HA-BDDE hydrogel system loaded with (ri)-r(P)ApoB_S_^Pro^. Upon coating, methicillin-resistant *S. aureus* (MRSA WKZ-2) and *E. coli* ATCC 25922 bacterial cells were plated into all the upper chambers. As shown in Figure 6c, only in the case of upper chambers coated with the hydrogel loaded with 320 μmol L^−1^ peptidomimetic, the migration of bacterial cells of both strains was found to be significantly attenuated.

### 3.8. Biocompatibility of HA-BDDE Hydrogel System Loaded with (ri)-r(P)ApoB_S_^Pro^ on Human Skin Cell Cultures

**Figure 7:**
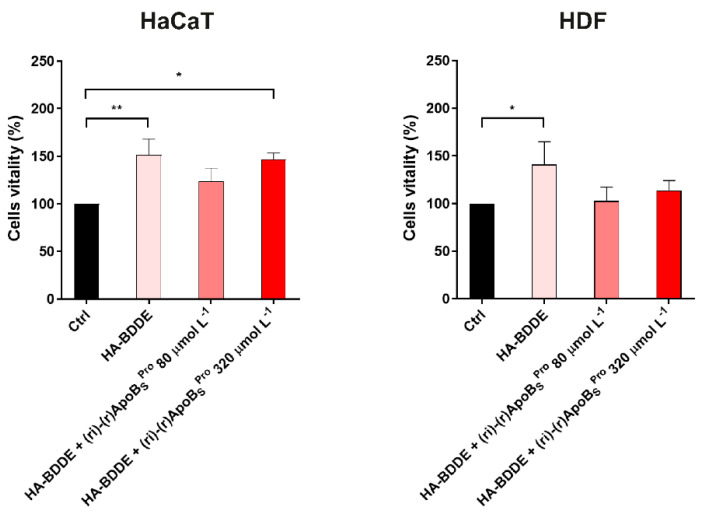
Effects of HA-BDDE hydrogel system functionalized with the peptidomimetic on human eukaryotic cell lines. Effects of HA-BDDE hydrogel system loaded with different concentrations of (ri)-r(P)ApoB_S_^Pro^ on the viability of HaCaT (immortalized human keratinocytes) and HDF (human dermal fibroblasts) cells. Cell viability was assessed by MTT assays and expressed as the percentage of viable cells compared to untreated cells (control). Experiments were performed three times. Error bars represent the standard deviation of the mean. Significant differences were indicated as * *p* < 0.05 or ** *p* < 0.01, for treated vs. control samples.

To evaluate the biocompatibility of HA-BDDE hydrogel system loaded with (ri)-r(P)ApoB_S_^Pro^ peptidomimetic toward human skin cell cultures, MTT assays were performed on immortalized human keratinocytes (HaCaTs) and on normal human fibroblasts (HDFs). Results indicated that HA-BDDE hydrogels loaded with 80 or 320 μmol L^−1^ (ri)-r(P)ApoB_S_^Pro^ did not affect the viability of HaCaT and HDF cell lines (Figure 7). A significant improvement of cell viability was, instead, detected when the cells were incubated with an unfunctionalized HA-BDDE hydrogel system or with HA-BDDE hydrogel loaded with 320 μmol L^−1^ peptidomimetic (Figure 7). This can be explained by considering that, as a major component of the extracellular matrix, hyaluronic acid plays a key role in skin repair mechanisms being able to enhance the proliferation and differentiation of endothelial cells and to facilitate cell migration during the wound healing process [46,47]. The overall results highlight the biocompatibility of the HA-BDDE hydrogel system loaded with the (ri)-r(P)ApoB_S_^Pro^ peptidomimetic and add an important aspect to the future applicability of this system.

## 4. Discussion

The incessant spreading of drug-resistant bacteria prompts the search for novel antibiotics and for novel materials able to efficiently deliver antimicrobial agents to the site of infection. In this scenario, the discovery of novel antimicrobials less prone to induce the development of resistance phenotype is urgently needed. Antimicrobial peptidomimetics are small molecules with evident advantages over conventional antibiotics, including a lower probability of inducing resistance development [3] and improved pharmacokinetic profiles [48]. The synthetic retro-inverso peptide (ri)-r(P)ApoB_S_^Pro^ was found to be more stable than its L-enantiomeric counterpart parental version [17] and to retain the broad-spectrum antimicrobial and anti-biofilm properties, thus being a good candidate to develop effective therapeutic strategies to treat recalcitrant infections. Importantly, the (ri)-r(P)ApoB_S_^Pro^ peptidomimetic not only is not toxic toward eukaryotic cells, but it also exhibits a protective effect, being able to reduce the number of bacterial cells infecting keratinocytes. In order to develop a suitable system to topically deliver the peptidomimetic, in this work, a hyaluronic acid (HA)–based hydrogel system was selected and tested. HA represents an ideal candidate, since it not only contributes to viscoelasticity and lubrication of tissues but also plays an important role in physiological processes, such as inflammation, wound healing, and tissue development [49]. These properties combined with the antimicrobial features of the peptidomimetic represent an optimal starting point to develop a feasible dermatological formulation [29]. The presence of effective concentrations of the peptidomimetic in the hydrogel system was found not to affect its physiochemical properties. Even more interestingly, the bioactive peptidomimetic was found to be rapidly released by the system. The anti-infective activity of the system was confirmed by assays on both Gram-negative and Gram-positive bacterial strains, including cells characterized by resistance to conventional antibiotics. Furthermore, the HA-hydrogel system loaded with (ri)-r(P)ApoB_S_^Pro^ was demonstrated to be able to counteract bacterial migration across surfaces, a property that opens further interesting perspectives in the treatment of dermal and epidermal infections. It has also to be highlighted that not only the HA-hydrogel system loaded with (ri)-r(P)ApoB_S_^Pro^ is biocompatible toward human skin cells, but the presence of hyaluronic acid is also responsible for a detectable improvement of keratinocytes viability, thus suggesting putative positive effects on skin wound healing. Altogether, these findings open novel and interesting perspectives to the applicability of (ri)-r(P)ApoB_S_^Pro^ in the treatment of skin infections and injuries. Further studies will be necessary in the future to optimize the herein developed hydrogel-based system and to test its efficacy in pre-clinical mouse models.

## Data Availability

Data is contained within the article.
